# Radiosensitivity in breast cancer assessed by the histone γ-H2AX and 53BP1 foci

**DOI:** 10.1186/1748-717X-8-98

**Published:** 2013-04-24

**Authors:** Cholpon S Djuzenova, Ines Elsner, Astrid Katzer, Eike Worschech, Luitpold V Distel, Michael Flentje, Bülent Polat

**Affiliations:** 1 Department of Radiation Oncology, University of Würzburg, Josef-Schneider-Str. 11, Würzburg D-97080, Germany; 2 Department of Radiation Oncology, University of Erlangen-Nürnberg, Universitätsstraße 27, Erlangen, D-91054, Germany

**Keywords:** DNA damage, DNA repair, Peripheral blood lymphocytes, Radiosensitivity

## Abstract

**Background:**

High expression of constitutive histone γ-H2AX, a sensitive marker of DNA damage, might be indicative of defective DNA repair pathway or genomic instability. 53BP1 (p53-binding protein 1) is a conserved checkpoint protein with properties of a DNA double-strand breaks sensor. This study explores the relationship between the clinical radiosensitivity of tumor patients and the expression/induction of γ-H2AX and 53BP1 *in vitro*.

**Methods:**

Using immunostaining, we assessed spontaneous and radiation-induced foci of γ-H2AX and 53 BP1 in peripheral blood mononuclear cells derived from unselected breast cancer (BC) patients (n=57) undergoing radiotherapy (RT). Cells from apparently healthy donors (n=12) served as references.

**Results:**

Non-irradiated cells from controls and unselected BC patients exhibited similar baseline levels of DNA damage assessed by γ-H2AX and 53BP1 foci. At the same time, the γ-H2AX assay of *in vitro* irradiated cells revealed significant differences between the control group and the group of unselected BC patients with respect to the initial (0.5 Gy, 30 min) and residual (2 Gy, 24 h post-radiation) DNA damage. The numbers of 53BP1 foci analyzed in 35 BC patients were significantly higher than in controls only in case of residual DNA damage. A weak correlation was found between residual foci of both proteins tested. In addition, cells from cancer patients with an adverse acute skin reaction (grade 3) to RT showed significantly increased radiation-induced γ-H2AX foci and their protracted disappearance compared to the group of BC patients with normal skin reaction (grade 0–1). The mean number of γ-H2AX foci after 5 clinical fractions was significantly higher than that before RT, especially in clinically radiosensitive patients.

**Conclusions:**

The γ-H2AX assay may have potential for screening individual radiosensitivity of breast cancer patients.

**Trial registration:**

http://www.krebshilfe.de/wir-foerdern.html

## Introduction

Breast cancer (BC) is the common type of tumor in females, accounting for approximately 21% of all cancer cases in women worldwide [1]. Among BC patients 2% have a strong genetic predisposition, caused by the mutations in highly penetrant *BRCA1* and *BRCA2* genes [2]. Because these genes cannot explain the overall increased risk in the relatives of BC cases [3], it was suggested that a substantial proportion of BC patients may be predisposed to cancer through mutations in low penetrance genes [4-6], which might be involved in DNA damage processing and repair.

Several DNA damage repair pathways constitute a guard system that protects cells against genetic instability and tumorigenesis. Both genetic instability and impaired DNA repair have been proposed as factors underlying increased susceptibility to tumorigenesis (for reviews, *see*[7,8]). The biological significance of genetic instability and DNA repair mechanisms in cancer development is particularly well illustrated by the autosomal recessive disorders, such as Ataxia telangiectasia, Fanconi anemia and Nijmegen breakage syndrome. These chromosome breakage syndromes are characterized by defects in DNA repair, predisposition to different forms of malignancy and increased radiosensitivity (for review, *see*[9]). Besides these rare syndromes, the deficient DNA repair capacity has been proposed to be a predisposing factor in familial BC and in some sporadic BC cases [10]. Genomic instability has also been described for various hereditary cancers including hereditary BC [3,11].

Genomic instability and DNA repair capacity have been analyzed in numerous population-based studies using a variety of assays that assess chromosomal aberrations, sister chromatid exchanges, micronuclei, DNA fragmentation by means of the Comet assay, etc. Some of these studies have revealed reduced DNA repair capacity in peripheral blood mononuclear cells (PBMCs, exposed *in vitro* to ionizing radiation (IR) or UV) from BC patients, as evaluated by the chromosome aberration assay [10,12,13] as well as by the micronucleus test [3,6,14]. In addition, histone γ-H2AX can serve as a further useful marker of DNA integrity and repair [15]. As shown by [16], constitutive expression of histone γ-H2AX may indicate disruption of the DNA damage repair pathway and/or genetic instability. In that study, the authors have observed a subset of patients with triple negative breast cancer based on γ-H2AX positivity in the formalin fixed tumor samples and embedded breast cancer cell lines [16].

Furthermore, the kinetics of formation or loss of γ-H2AX foci might be related to the efficiency of “repair” of higher order chromatin organization [17]. By counting γ-H2AX foci in blood lymphocytes, Rübe et al. (2010) [18] found impaired DNA repair capacity in cells from children with tumors [18]. At the same time, the initial numbers of γ-H2AX foci after *in vitro* irradiation were found very similar among the groups studied [18]. Moreover, Brzozowska et al. (2012) [19] found, by flow cytometry, an increased expression of histone γ-H2AX in irradiated blood lymphocytes from normal donors, as compared to tumor patients [19]. But the difference was not confirmed when γ-H2AX foci were counted by fluorescence microscopy. A series of studies [18-24] used histone γ-H2AX as a marker to predict the toxicity in normal tissue during radiotherapy of tumor patients, however, with contradictory outcomes. Thus, some of the quoted studies [19,21-23] revealed no correlation between either acute or late side effects of radiation therapy (RT) and expression of histone γ-H2AX. In contrast, other workers [18,20] found that the disappearance of histone γ-H2AX correlated with high-grade toxicity from RT treatment. Henríquez-Hernández et al. (2011) [24] also suggest that lower levels of initial DNA damage may be associated with a lower risk of suffering from severe late subcutaneous toxicity [24].

Despite extensive studies into the relationship between cellular radiation tests, cancer risk and clinical radiation reaction, a general opinion has not yet been formed. The discrepancies cited above prompted us to explore whether the histone γ-H2AX assay is able to predict the clinical radiation reaction of BC patients and to discriminate them from healthy subjects. We examined both baseline and radiation-induced DNA damage in PBMCs from a group of 57 unselected BC patients compared with a group of healthy subjects (n=12). PBMCs from a small group (n=6) of BC patients with an adverse early skin reaction to radiotherapy have also been retrospectively analyzed. In addition to γ-H2AX, we analyzed the foci of 53BP1 (p53-binding protein 1), a well-known sensor protein of DNA damage [25]. DNA double-strand breaks (DSB) attract the 53BP1 protein to the surrounding chromatin, where the 53BP1 is recruited by methylated H3 Lys 79 and signals chromatin/DNA damage [25] in a γ-H2AX-dependent manner.

## Materials and methods

### Subjects

The assay was performed on PBMCs isolated from two groups of individuals: (1) a group (n=57) of unselected BC patients who were prospectively involved in the study and their blood samples were collected before and during (72 h after 5 clinical fractions) clinical radiation; and (2) a group of apparently healthy donors (n=12), mainly hospital personal. To our knowledge, none of the healthy controls was previously exposed to radiation. All patients and healthy donors were asked to complete a questionnaire on their medical histories and lifestyles, including genetic diseases, medication, alcohol consumption, smoking (Additional file 1: Tables S1 and S2). Tumor stage (Additional file 1: Table S1) was determined according to the standard TNM criteria. This evaluation yielded 3, 37, 15, 1, and 1 case(s) scored as stage DCIS, T1, T2, T3, and T4, respectively (Additional file 1: Table S2). Tumor patients receiving chemotherapy during RT were excluded. The study was approved by the University of Würzburg Ethics Committee and all patients and donors gave informed written consent.

RT treatment of cancer patients was performed by means of a 6 MV linear accelerator (Siemens Concord, CA, USA) at a dose rate of 2 Gy/min. All BC patients received a tangential irradiation of the whole breast, with lateral and medial wedge fields. The regimen comprised a total dose of 50 or 60 Gy with a fractionation dose of 2 Gy five times a week. The early skin reaction to RT developing in the skin within the radiation field of the breast was controlled at the end of RT and used as an indicator for clinical radiosensitivity according RTOG score [26].

### Blood sampling and isolation of cells

PBMCs were separated from the heparinized blood samples by density-gradient centrifugation using Ficoll-Histopaque 1077 (Sigma 1077–1, Deisenhofen, Germany) according to the manufacturer's instructions. PBMCs were washed twice with Ca^2+^- and Mg^2+^-free physiological phosphate-buffered saline (PBS, Sigma D-8537) and finally resuspended in the RPMI 1640 (Sigma R-8758) supplemented with 10% FBS, glutamine (1 mM), and penicillin-streptomycin (100 U/ml and 100 μg/ml, respectively), hereafter denoted as complete growth medium (CGM), and incubated at 37°C in a humidified atmosphere enriched with 5% CO_2_ until irradiation.

### In vitro X-ray irradiation

The final cell density of isolated PBMCs was adjusted to 1 × 10^6^ cells/ml and the samples were placed at 37°C in a 5% CO_2_ incubator. X-irradiation (0.5 and 2 Gy) was performed using a 6 MV Siemens linear accelerator (Siemens Concord, CA, USA) at a dose rate of 2 Gy/min. Non-irradiated cells were treated in similar way, but at a zero radiation dose.

### Immunofluorescence staining

A cell aliquot (2–3 × 10^5^) of control or irradiated cells was cytocentrifuged at various time points after IR on a glass slide and fixed for 15 min in ice-cold methanol, and then for 1 min in 100% acetone at -20°C. Slides were washed three times for 5 min in PBS and blocked with 4% FBS-PBS for 1 h at room temperature [27]. Blindly coded slides were incubated overnight at 4°C with either anti-phospho-histone H2AX (Millipore, Schwalbach, Germany, # 05–636), or anti-53BP1 (Novus Biologicals, Cambridge, UK, NB 100–304) antibodies followed by incubation with respective secondary antibodies conjugated with Alexa Fluor 488 or 594 nm. Slides were counterstained with 0.2 μg/ml of DAPI (4’,6’-diamidino-2-phenylindole) in antifade solution (1.5% N-propyl-gallate, 60% glycerol in PBS) and examined using a Leica DMLB epifluorescence microscope (at a 1000*x* magnification) coupled to a cooled CCD camera (ColorView 12, Olympus Biosystems, Hamburg, Germany). Camera control and image acquisition were done with image analysis software (Olympus Biosystems, Hamburg, Germany). The foci were counted by eye in 500 cells per each treatment condition, no threshold for γ-H2AX or 53BP1 was set. The cells with apoptotic morphologies or cells with bright nuclei (intense, complete coverage of the nuclei with foci staining) were excluded from the analyses. Because the wide-field microscopic setup used here does not allow three-dimensional microscopy with Z-planning, two-dimensional images were captured from the focal plane.

### Statistics

Data are presented as mean (± SD). Mean values were compared by the Student's *t*-test or one way ANOVA. The threshold of statistical significance was set at p < 0.05. Statistics was performed with the program Origin 8.5 (Microcal, Northampton, MS, USA).

## Results

**Figure 1:**
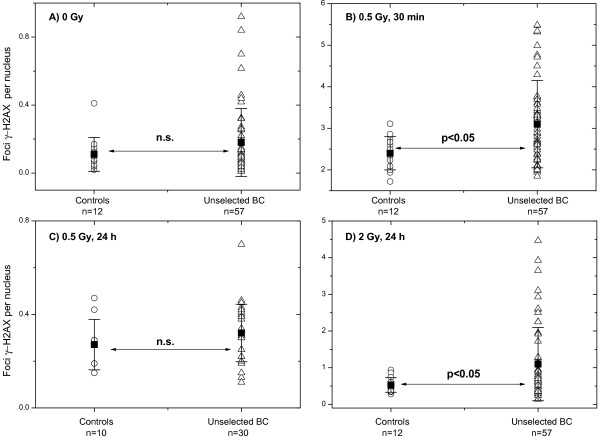
**Comparison of histone γ-H2AX foci in PBMCs derived from control donors and unselected BC patients. **DNA damage assessed by means of the histone γ-H2AX assay in non-irradiated **(A) **and in irradiated **(B-D) **PBMCs derived from unselected BC patients (triangles), as compared to the cells from apparently healthy donors (circles). Initial (**B**), residual (C - 0.5 Gy, 24 h, D - 2 Gy, 24 h) DNA damage were assessed in PBMCs after irradiation with 0.5 Gy (**B**, **C**) or 2 Gy (**D**) *in vitro*. Filled squares represent the mean values (± SD) for the respective group. “n.s.” indicates that the difference was not highly significant (p > 0.05).

The damage to DNA and its repair kinetics were evaluated up to 24 h after exposure to 0.5 Gy or 2 Gy of X-rays *in vitro*. The extent of DNA damage was assessed by counting the number of histone γ-H2AX foci, a sensitive marker of DNA double-strand breaks (DSBs) [28]. The mean data from 500 nuclei were determined for the cell samples from each tested individual (Figure 1). The means for each tested group of individuals are also shown in Figure 1.

The data on initial, residual and background DNA damage measured by histone γ-H2AX for each individual, as well as the age, sex and grade of skin reaction to RT are given in Table 1, from which the following trends are obvious (*see* also Figure 1). Although non-irradiated cells of some cancer patients exhibited noticeably lower baseline amounts of DNA damage, i.e. in the range of controls, the *mean* value of background DNA damage (Figure 1A) was higher (0.2±0.2 foci/nucleus) in the BC patient group, as compared to the group of healthy individuals (0.1±0.1). However, the difference does not reach statistical significance, which may be in part due to the strong interindividual variability. In contrast, after irradiation *in vitro*, the γ-H2AX assay did reveal significant differences between the two groups in terms of their initial (Figure 1B, 0. 5 Gy, 30 min) and residual levels of DNA damage (Figure 1D, 2 Gy, 24 h).

**Table 1:** DNA damage measured by the histone γ-H2AX in PBMCs isolated from blood of apparently healthy donors (N) and unselected breast cancer (BC) patients after exposure to 0.5 or 2 Gy of X-irradiation in vitro* or after 5 clinical fractions (BC patients)

**Subject**^**a**^	**Age (years)**	**Sex**	**Clinical status with respect to cancer, skin reaction to RT**^**b**^	**0 Gy**	**0.5 Gy, 30’**	**0.5 Gy, 24 h**	**2 Gy, 24 h**	**72 h after 5 clinical fractions**
**Apparently healthy donors**								
N-1	27	F	n.d.	0.02	3.11		0.94	
N-2	28	F	n.d.	0.04	2.60		0.86	
N-3	29	F	n.d.	0.13	1.94	0.29	0.55	
N-4	58	F	n.d.	0.07	2.00	0.28	0.41	
N-5	58	F	n.d.	0.15	2.86	0.47	0.39	
N-6	65	F	n.d.	0.12	2.73	0.42	0.74	
N-7	56	F	n.d.	0.05	1.72	0.15	0.28	
N-8	48	F	n.d.	0.41	2.67	0.28	0.60	
N-9	41	F	n.d.	0.17	2.49	0.29	0.37	
N-10	44	F	n.d.	0.07	2.09	0.19	0.34	
N-11	48	F	n.d.	0.08	2.33	0.15	0.47	
N-12	43	F	n.d.	0.10	2.23	0.19	0.36	
Mean	45			0.12	2.40	0.27	0.53	
± SD	12			0.10	0.40	0.10	0.21	
**Breast cancer patients**								
BC-01	59	F	**BC, grade 3**	0.70	3.29		2.59	0.32
BC-02	63	F	BC, grade 1	0.62	2.39		2.93	0.57
BC-03	45	F	BC, grade 0	0.92	2.87		3.93	0.67
BC-04	73	F	**BC, grade 3**	0.42	4.50		2.61	0.66
BC-05	57	F	BC, grade 1	0.07	5.48		2.50	0.49
BC-06	50	F	BC, grade 1	0.27	4.77		1.72	0.56
BC-07	63	F	BC, grade 2	0.46	5.32		3.65	0.75
BC-08	70	F	BC, grade 2	0.84	5.49		4.47	0.64
BC-12	61	F	**BC, grade 3**	0.25	4.71		2.26	0.57
BC-13	55	F	**BC, grade 3**	0.32	5.34		3.11	0.83
BC-14	54	F	BC, grade 2	0.14	3.75		1.18	0.19
BC-20	52	F	BC, grade 1	0.32	3.77		1.95	1.06
BC-23	51	F	BC, grade 1	0.12	2.67		2.25	0.77
BC-25	41	F	BC, grade 0	0.44	4.99		1.27	0.30
BC-26	63	F	BC, grade 1	0.21	3.62		1.92	0.35
BC-27	71	F	BC, grade 1	0.14	3.31		0.92	0.57
BC-28	42	F	BC, grade 0	0.10	4.29		1.29	0.35
BC-29	74	F	BC, grade 1	0.22	3.7		1.12	0.28
BC-30	46	F	BC, grade 2	0.05	3.39		0.77	0.32
BC-31	56	F	BC, grade 2	0.01	1.95		0.95	0.27
BC-32	44	F	BC, grade 2	0.08	2.98		1.08	0.28
BC-33	41	F	**BC, grade 3**	0.19	3.43		0.92	0.29
BC-34	38	F	BC, grade 2	0.10	3.22		0.86	0.28
BC-35	77	F	BC, grade 2	0.07	3.23		0.91	0.06
BC-36	70	F	BC, grade 1	0.06	2.72		0.81	0.18
BC-37	64	F	BC, grade 0	0.03	2.90		0.48	0.19
BC-38	73	F	BC, grade 1	0.08	2.37		0.48	0.20
BC-39	55	F	BC, grade 1	0.01	2.05	0.13	0.31	0.43
BC-40	77	F	BC, grade 2	0.05	2.24	0.11	0.15	0.34
BC-41	54	F	BC, grade 1	0.09	2.11	0.31	0.39	0.38
BC-42	49	F	BC, grade 1	0.01	2.05	0.15	0.40	0.34
BC-43	62	F	BC, grade 2	0.05	1.99	0.43	0.62	0.57
BC-44	59	F	BC, grade 1	0.04	2.54	0.41	0.75	0.18
BC-45	51	F	BC, grade 1	0.02	2.81	0.40	0.64	0.24
BC-46	62	F	BC, grade 1	0.32	3.58	0.70	0.28	0.32
BC-47	30	F	BC, grade 2	0.22	3.27	0.41	0.24	0.18
BC-48	71	F	BC, grade 2	0.26	3.48	0.46	0.59	0.51
BC-49	46	F	BC, grade 2	0.18	3.15	0.38	0.60	0.30
BC-50	48	F	**BC, grade 3**	0.13	3.34	0.33	0.31	0.34
BC-51	57	F	BC, grade 1	0.09	2.63	0.39	0.34	0.41
BC-52	62	F	BC, grade 2	0.09	2.54	0.25	0.36	0.31
BC053	68	F	BC, grade 2	0.09	2.23	0.20	0.34	0.16
BC-54	37	F	BC, grade 1	0.14	2.28	0.25	0.47	0.4
BC-55	67	F	BC, grade 1	0.08	2.73	0.41	0.4	0.27
BC-57	55	F	BC, grade 1	0.11	2.68	0.3	0.76	0.38
BC-58	62	F	BC, grade 1	0.06	2.62	0.31	0.6	0.32
BC-59	55	F	BC, grade 1	0.07	1.85	0.22	0.41	0.36
BC-60	65	F	BC, grade 2	0.11	3.32	0.42	0.54	0.43
BC-61	54	F	BC, grade 2	0.04	2.09	0.25	0.52	0.43
BC-62	53	F	BC, grade 2	0.06	0.06	0.19	0.14	0.38
BC-63	69	F	BC, grade 1	0.09	2.65	0.34	0.59	0.4
BC-64	81	F	BC, grade 1	0.1	2.65	0.39	0.36	0.27
BC-65	38	F	BC, grade 2	0.15	2.01	0.31	0.67	0.31
BC-66	77	F	BC, grade 1	0.14	2.29	0.3	0.64	0.27
BC-67	48	F	BC, grade 1	0.14	2.61	0.45	0.71	0.36
BC-68	49	F	BC, grade 2	0.1	2.48	0.22	0.53	0.27
BC-69	51	F	BC, grade 1	0.26	2.25	0.2	0.53	0.16
Mean	57			0.18	3.07	0.32	1.11	0.39
± SD	12			0.19	1.04	0.12	1.01	0.19

*Each indicated DNA damage parameter represents the mean value obtained on the cells from a given individual. Bold fonts indicate the unselected BC patients who revealed an adverse acute skin reaction to RT;

^**a**^Case number was according to our files;

^**b**^Early skin reaction according RTOG score (Cox et al., 1995) [26]. RTOG grade: 1 - follicular, faint or dull erythema, dry desquamation; 2 - tender or bright erythema, moderate edema; 3 - confluent, moist desquamation, pitting edema; 4 - ulceration, haemorrhage, necrosis.

Abbreviations used: N, normal; BC, breast cancer; F, female; n.d., not determined.

**Figure 2:**
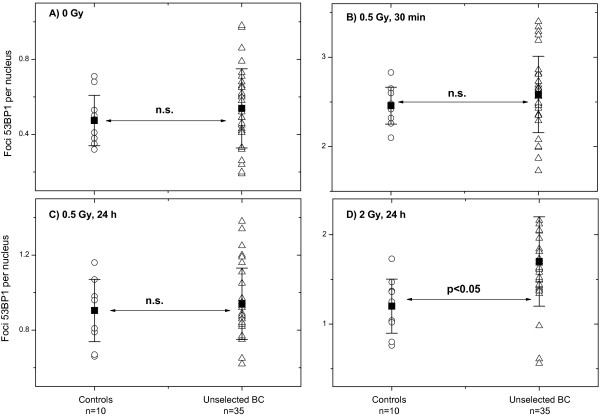
**Comparison of 53BP1 foci in PBMCs derived from control donors and unselected BC patients. **DNA damage assessed by the mean number of 53BP1 foci in non-irradiated **(A)** and irradiated **(B-D) **PBMCs derived from unselected BC patients (triangles), as compared to cells from apparently healthy donors (circles). For further details, *see *legend to Figure 1.

In addition, the foci numbers of 53BP1, a sensor of DNA damage [25], were compared between 10 healthy samples and 35 samples from BC patients (Table 2). As seen in Figure 2, the mean expression levels of this marker protein were very similar in two groups, except for the time point of 24 h after irradiation with 2 Gy. As suggested by a reviewer, we compared the expression of γ-H2AX and 53BP1 per nucleus at different time and radiation doses. Judging from the correlation coefficients given in Additional file 1: Figure S1, there was a weak correlation between residual amounts of γ-H2AX and 53BP1 foci (Additional file 1: Figure S1, parts C and D), but no correlation (Additional file 1: Figure S1, parts A and B) was found between background and induced (0.5 Gy, 30 min) γ-H2AX and 53BP1 foci.

**Table 2:** DNA damage measured by 53BP1 in PBMCs isolated from blood of apparently healthy donors (N) and unselected breast cancer (BC) patients after exposure to 0.5 or 2 Gy of X-irradiation in vitro* or after 5 clinical fractions (BC patients)

**Subject**^**a**^	**Age (years)**	**Sex**	**Clinical status with respect to cancer, skin reaction to RT**^**b**^	**0 Gy**	**0.5 Gy, 30’**	**0.5 Gy, 24 h**	**2 Gy, 24 h**	**72 h after 5 clinical fractions**
**Apparently healthy donors**								
N-3	29	F	n.d.	0.71	2.47	0.99	1.47	
N-4	58	F	n.d.	0.68	2.83	0.96	1.37	
N-5	58	F	n.d.	0.41	2.65	1.16	1.25	
N-6	65	F	n.d.	0.35	2.48	1.07	1.73	
N-7	56	F	n.d.	0.53	2.26	0.66	0.76	
N-8	48	F	n.d.	0.38	2.32	0.98	1.36	
N-9	41	F	n.d.	0.32	2.10	0.96	1.04	
N-10	44	F	n.d.	0.50	2.46	0.79	1.02	
N-11	48	F	n.d.	0.48	2.60	0.81	1.20	
N-12	43	F	n.d.	0.38	2.42	0.67	0.80	
Mean	45			0.47	2.46	0.91	1.20	
± SD	12			0.13	0.20	0.16	0.29	
**Breast cancer patients**								
BC-01	59	F	**BC**, **grade 3**	0.13	0.85	n.d.	1.84	1.73
BC-04	73	F	**BC**, **grade 3**	0.15	1.24	n.d.	2.61	1.68
BC-12	61	F	**BC**, **grade 3**	0.11	1.19	n.d.	2.18	0.95
BC-13	55	F	**BC, grade 3**	0.35	2.47	n.d.	2.86	1.23
BC-33	42	F	**BC**, **grade 3**	0.35	0.87	n.d.	2.78	1.60
BC-39	55	F	BC, grade 1	0.32	2.56	0.75	1.41	0.90
BC-40	77	F	BC, grade 2	0.61	2.43	0.62	0.61	0.78
BC-41	54	F	BC, grade 1	0.97	2.36	1.11	1.34	0.91
BC-42	49	F	BC, grade 1	0.19	2.48	0.76	1.68	0.80
BC-43	62	F	BC, grade 2	0.33	1.99	0.92	1.58	0.42
BC-44	59	F	BC, grade 1	0.24	2.08	1.25	2.16	0.58
BC-45	51	F	BC, grade 1	0.20	1.73	1.20	2.12	0.77
BC-46	62	F	BC, grade 1	0.33	2.73	1.34	0.56	0.74
BC-47	30	F	BC, grade 2	0.26	2.57	1.38	0.98	0.65
BC-48	71	F	BC, grade 2	0.79	3.34	1.16	1.96	0.93
BC-49	46	F	BC, grade 2	0.49	3.19	1.19	1.84	0.62
BC-50	48	F	**BC**, **grade 3**	0.68	3.29	0.88	1.39	0.68
BC-51	57	F	BC, grade 1	0.71	2.82	0.93	1.43	0.50
BC-52	62	F	BC, grade 2	0.66	2.48	0.87	1.50	0.59
BC-53	68	F	BC, grade 2	0.98	2.81	0.84	1.37	0.55
BC-54	37	F	BC, grade 1	0.65	2.29	0.84	1.49	0.95
BC-55	67	F	BC, grade 1	0.73	2.35	0.89	1.50	0.71
BC-57	55	F	BC, grade 1	0.86	3.40	0.89	2.04	0.69
BC-58	62	F	BC, grade 1	0.52	3.25	0.95	2.05	0.57
BC-59	55	F	BC, grade 1	0.45	2.69	0.82	1.62	0.88
BC-60	65	F	BC, grade 2	0.65	2.86	0.97	1.61	0.49
BC-61	54	F	BC, grade 2	0.54	2.66	0.88	1.63	0.96
BC-62	53	F	BC, grade 2	0.49	2.65	0.77	1.37	0.72
BC-63	69	F	BC, grade 1	0.62	2.73	1.05	1.81	0.58
BC-64	81	F	BC, grade 1	0.6	2.62	0.84	1.37	0.47
BC-65	38	F	BC, grade 2	0.58	2.35	0.95	1.70	0.87
BC-66	77	F	BC, grade 1	0.46	2.46	0.83	1.44	0.57
BC-67	48	F	BC, grade 1	0.43	1.87	0.86	1.52	0.66
BC-68	49	F	BC, grade 2	0.42	2.49	0.65	1.73	0.73
BC-69	51	F	BC, grade 1	0.41	2.00	0.84	1.70	0.49
Mean	57			0.49	2.40	0.94	1.70	0.80
± SD	12			0.23	0.63	0.19	0.49	0.32

*Each indicated DNA damage parameter represents the mean value obtained on the cells from a given individual. Bold fonts indicate the unselected BC patients who revealed an adverse acute skin reaction to RT;

^**a**^Case number was according to our files;

^**b**^Early skin reaction according RTOG score (Cox et al., 1995) [26]. RTOG grade: 1 - follicular, faint or dull erythema, dry desquamation; 2 - tender or bright erythema, moderate edema; 3 - confluent, moist desquamation, pitting edema; 4 - ulceration, haemorrhage, necrosis.

Abbreviations used: N, normal; BC, breast cancer; F, female; n.d. indicates not determined.

**Figure 3:**
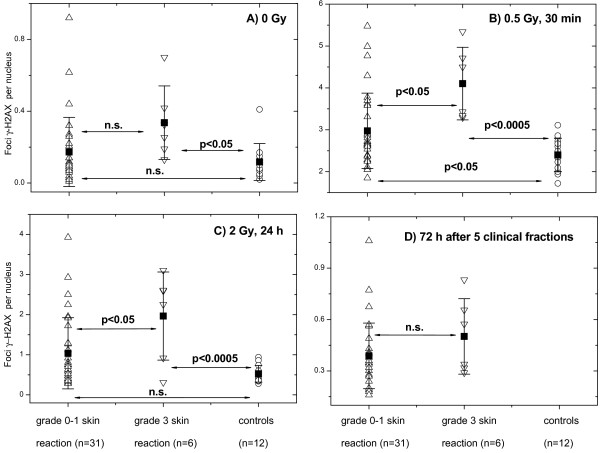
**Histone γ-H2AX foci in PBMCs derived from normally-reacting and radiosensitive BC patients compared with control donors. **DNA damage assessed by means of the histone γ-H2AX assay in non-irradiated **(A) **and irradiated **(B-D) **PBMCs derived from normally-reacting BC patients (up triangles) and radiation-sensitive (down triangles) cancer patients compared to cells from apparently healthy donors (circles). Initial (**B**), residual DNA damage 24 h after *in vitro *2 Gy (**C**) or 72 h after 5 clinical radiation fractions (**D**) were assessed in PBMCs after irradiation with *in vitro *(**B**, **C**) or *in vivo *(**D**). Filled squares represent the mean values (± SD) for the respective group. “n.s.” indicates that the difference was not highly significant (p > 0.05).

Out of 57 prospectively recruited BC patients, six exhibited an increased early skin reaction to RT (grade 3 according RTOG score, *see* Table 1, cases BC01, BC04, BC12, BC13, BC33 and BC48). Based on the clinical skin reaction of radiotherapy patients we analyzed retrospectively the initial, residual and background DNA damage measured by histone γ-H2AX between the groups of BC patients with normal (RTOG grade 0 and 1, n=31) and an adverse (RTOG grade 3, n=6) skin reaction to RT compared with the healthy donors (Figure 3). As seen in Figure 3A, background DNA damage in PBMCs from BC patients with increased clinical skin reaction was higher than that from control donors. The induced DNA damage 30 min after *in vitro* application of 0.5 Gy (Figure 3B) and the residual DNA damage (Figure 3C, 2 Gy, 24 h) were higher in both BC groups than that in the cells from controls. However, blood samples taken from BC patients 72 h after the fifth clinical radiation dose showed mostly similar histone γ-H2AX expression in both groups of patients (Figure 3D).

**Figure 4:**
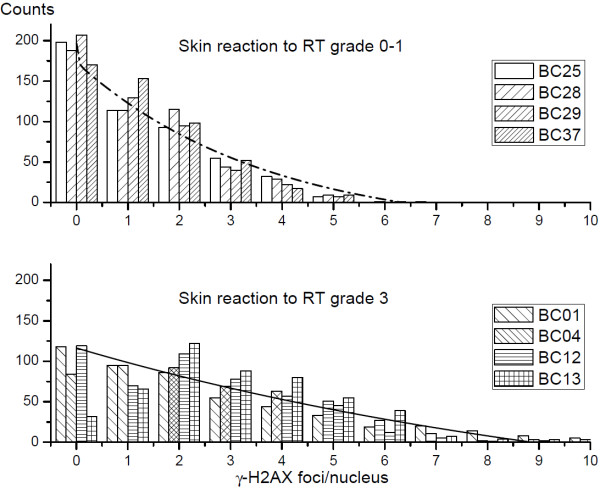
**Foci γ-H2AX distribution in PBMCs derived from normally-reacting and radiosensitive BC patients. **Histograms depicting the γ-H2AX foci distributions within cell samples from cancer patients with normal (grade 0–1, top histograms) and an adverse (grade 3, bottom histograms) skin reaction to RT. Cells were analyzed for γ-H2AX focus 24 h after irradiation with 2 Gy *in vitro*. Five hundreds nuclei were analyzed per each sample.

In order to elucidate the observed increase in the mean numbers of γ-H2AX foci in cells derived from hypersensitive cancer patients, we further analyzed the distributions of γ-H2AX foci within different cell samples. Figure 4 shows exemplarily the histograms of γ-H2AX foci per nucleus in cells from BC patients with normal (BC-25, BC-28, BC-57, top histograms) and adverse (BC-01, BC-04, BC-12, BC-13, bottom histograms) clinical sensitivity to RT. The PBMCs taken before clinical RT were irradiated *in vitro* with 2 Gy and allowed to repair 24 h post-IR, fixed and stained for histone γ-H2AX. We found that the fraction of γ-H2AX foci-negative cells in samples from normal sensitive BC patients was about 40%, whereas only 14% of cells were γ-H2AX negative in the samples from hypersensitive BC patients at this time point. The mean number of foci per nucleus in the samples from normally-reacting BC patients was about 1.0. Compared to the cells from normally-reacting BC patients, the histograms of cells from clinically sensitive BC patients were shifted to higher values, showing in average of about 2–3 foci per nucleus.

**Figure 5:**
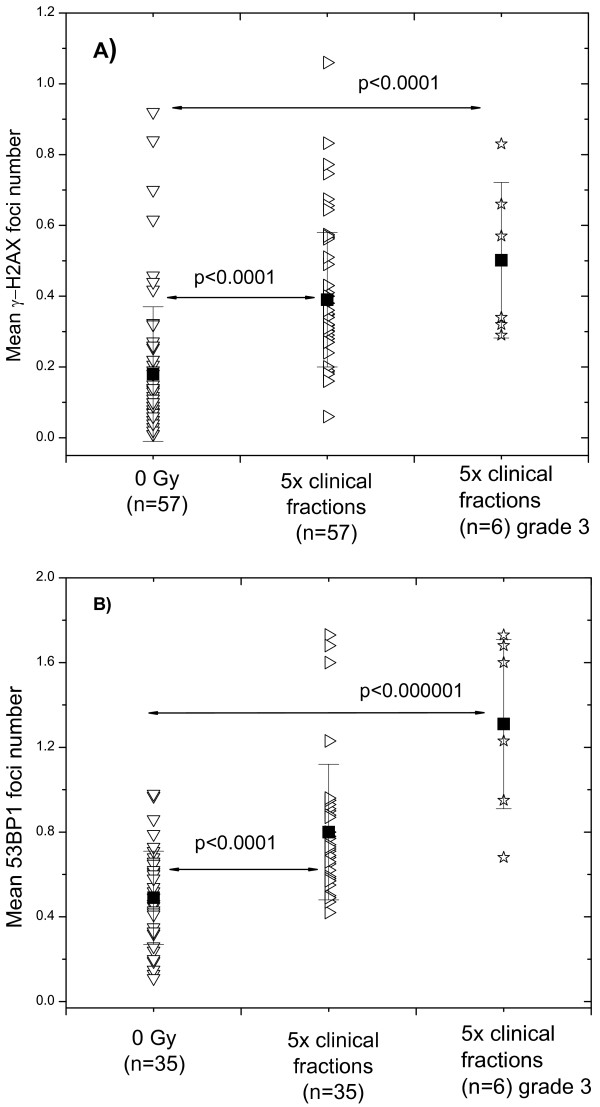
**Effect of clinical radiation on the expression of γ-H2AX and 53BP1 foci in blood lymphocytes. **DNA damage assessed by means of the histone γ-H2AX (A) and 53BP1 (B) assays in non-irradiated (down triangles) and in clinically irradiated (right triangles) PBMCs derived from unselected BC patients compared with BC patients with an adverse (grade 3) skin reaction to RT (asterisks). Filled squares represent the mean values (± SD) for the respective group.

In addition, as mentioned in the Materials and Methods, blood samples were withdrawn from all recruited BC patients after 5 clinical fractions and analyzed for γ-H2AX and 53BP1 foci. As seen in Figure 5A, the mean number of γ-H2AX foci per patient’s sample after 5 clinical fractions was significantly higher (0.39±0.19) than before RT (0.18±0.19). A group of BC patients with adverse skin reaction to RT showed an even higher number of γ-H2AX foci (0.50±0.22).

The quantification of 53BP1 foci (Figure 5B) was conducted in a smaller patients’ group (n=35 *vs*. n=57 tested for γ-H2AX), which however, contained all clinically sensitive BC patients (n=6) who showed an adverse skin reaction to RT. Comparison of the mean number of 53BP1 foci per patient’s sample after 5 clinical fractions revealed a significantly increased foci numbers after clinical irradiation (0.8±0.3 *vs*. 0.5±0.2 before RT) for the whole group tested (n=35). A subset of BC patients with adverse skin reaction to RT showed much higher number of 53BP1 foci (1.3±0.4) after 5 clinical fractions.

As suggested by a referee, we performed correlation analysis between tumor stage and the expression of γ-H2AX and 53BP1 foci. As seen in Additional file 1: Figure S2, tumor stages DCIS, T1 and T2 exhibited similar levels of expression of both γ-H2AX and 53BP1. In case of the advanced tumor stages T3 and T4, each counted only once, no statistical analysis could be done.

## Discussion

In this study, peripheral blood cells isolated from (1) unselected BC patients, and (2) healthy individuals were analyzed for their DNA damage using the histone γ-H2AX assay. During or after RT a small group of patients (n=6) showed an adverse early skin reaction to RT. The analysis of non-irradiated cell samples did not reveal any differences in the background DNA damage levels between the group of healthy donors and unselected BC patients (Figure 1A). However, if the radiotherapy patients’ group was split into the subgroups according to their acute clinical skin reaction (RTOG), the background DNA damage in cells derived from hypersensitive BC patients was significantly higher than that in healthy donors (Figure 3A). Irradiation *in vitro* of the samples from BC patients revealed an increased (p < 0.05) amount of γ-H2AX foci 30 min (Figure 1B, 0.5 Gy) and 24 h post-IR (Figure 1D, 2 Gy). The differences in γ-H2AX foci amounts 30 min and 24 h post-IR with 0.5 Gy and 2 Gy, respectively became even higher (p<0.0005) if the groups of healthy donors and the patients with an adverse clinical reaction to RT (Figure 3B, 3C) were compared. In addition, the mean background value of γ-H2AX in the samples of hypersensitive BC patients was significantly higher (p<0.05) than in control group. Interestingly, retrospective analysis of the groups of BC patients with normal (n=31) and an adverse (n=6) clinical reaction to RT revealed statistically significant differences in the induction (Figure 3B) and repair (Figure 3C) of DNA damage 30 min and 24 h post-IR with 0.5 and 2 Gy *in vitro*.

Our results disagree with those of Vasireddy et al. (2010) [23] who have found no differences in levels of both basal and radiation-induced DNA damage in cells from tumor patients with increased clinical radiosensitivity and healthy controls [23]. The reasons for the discrepancy might reside in the patients’ and controls’ cohorts, cancer stage, treatment prior to blood sampling, arbitrary determined cut-off values, experimental protocols, methods of foci quantification (flow cytometry *vs.* fluorescence microscopy) as well as in interlaboratory variability. Moreover, in contrast to the present and several other studies [18,20,21], which analyzed primary PBMCs or T-cells [19], the paper of Vasireddy et al. (2010) [23] used lymphoblastoid cell lines (LCLs) derived from cells of tumor patients [23]. Besides this, the quantification of histone γ-H2AX foci by fluorescence microscopy appears to differ greatly between laboratories. Thus, the background values of about 0.07-0.08 γ-H2AX foci pro lymphocyte in non-irradiated cells reported by Fleckenstein et al. (2012) [21] are some 3 times lower than the values presented here in Figure 1A.

As mentioned before, we found the differences in the induction and disappearance of γ-H2AX foci between the groups of patients with an adverse skin reaction to RT and normally-reacting patients or healthy donors (Figure 3B, 3C). These results disagree with the findings of some studies [19,20] who observed, by flow cytometry, a higher fluorescence of histone γ-H2AX in irradiated cells from healthy donors, as compared to the patients with severe side effects after RT. At the same time, the protracted rate of disappearance of histone γ-H2AX foci found in our study in cells of radiation-sensitive cancer patients corroborate well the data of Bourton et al. (2011) [20]. It may be argued that in the present study the small control group (an average age of 45±12 years) was younger than the group of BC patients (mean age of 57±12 years). The literature on age dependence of γ-H2AX formation, however, seems quite controversial. Based on the comparison of two donors groups differing markedly in age (31–45 *vs*. 50–72 years), Firsanov et al. (2011) [29] conclude that the dynamics of γ-H2AX formation is independent of age [29]. In contrast, Sedelnikova et al. (2008) found [30], by comparing two groups with a much larger deviation (21–30 years *vs*. 60–72 years) in age than our groups, that the portions (about 30%) of cells containing γ-H2AX foci in older individuals (60–72 years) was higher than in younger individuals (about 20%). But the incidence of γ-H2AX foci in response to IR was found to be age independent [30].

Since fortunately only a minority (about 5%) of RT patients develop either acute or late radiotoxic responses during or after RT [31], a cohort of 57 prospectively recruited BC patients in our study was too small to reveal several radiation-sensitive patients. Among them, we observed a small group (n=6) of patients exhibiting early skin radiotoxicity during or shortly after RT. Even so, there was a correlation of radiation response *in vivo* with increased initial and/or residual DNA damage level after irradiation *in vitro*, as well as with increased background DNA damage in a γ-H2AX assay compared to healthy controls. Furthermore, we found differences in the initial and residual DNA damage between irradiated cells from tumor patients with normal and those with an adverse clinical sensitivity to RT (Figure 3B, 3C, first and second data sets). In addition, samples derived from BC patients after 5 clinical radiation fractions showed an increased amount of γ-H2AX foci compared with that obtained before RT (Figure 5).

The second marker of DNA DSB formation studied here was the 53BP1 protein. Given that the γ-H2AX test reveals a DSB-induced protein modification and the 53BP1 foci indicate the accumulation of a DSB-modified chromatin protein [25,32], both types of radiation-induced foci should be very similar at all measurement points and mostly overlapping in fluorescence images [33]. In our hands, however, the 53BP1 assay was less sensitive than that for histone γ-H2AX in case of endogeneous (0 Gy) and induced (0.5 Gy, 30 min) foci. There may be at least two reasons for the observed discrepancy between two assays. Firstly, the quantification of γ-H2AX and 53BP1 foci was done in partially overlapping but different patients’ cohorts. Secondly, for detection of γ-H2AX and 53BP1 we used commercially available antibodies which were very different in their immunological properties, *i.e*. highly specific monoclonal antibodies against γ-H2AX and less selective polyclonal antibodies against 53BP1. Therefore, it cannot be excluded that if both antibody preparations were of the same specificity the tests would yield similar results. Besides this, the 53BP1 analysis was done for a much smaller patient’s group (n=35), as compared to γ-H2AX assay (n=57). Nevertheless, residual (24 h post-IR) foci of both proteins were found to be weakly correlated (Figure S1).

In conclusion, prospectively recruited BC patients showed on average increased initial and residual DNA damage levels measured by histone γ-H2AX, as compared with the healthy group. However, due to a large interindividual variability, it was not possible to discriminate BC patients from healthy individuals. But a minor (n=6) group of retrospectively identified BC patients with an adverse skin reaction to RT (analyzed separately from normally-reacting BC patients), showed striking differences by the γ-H2AX assay with respect to healthy individuals. The γ-H2AX assay was also able to identify BC patients with normal clinical reaction to RT on the basis of the induced and residual DNA damage, but not on the basis of background DNA damage. A larger study would be necessary in order to investigate the complex mechanisms behind the normal tissue radiotoxicity.

## Abbreviations

BC: Breast cancer; IR: Ionizing radiation; PBS: Phosphate buffered saline; PBMCs: Peripheral blood mononuclear cells; RT: Radiotherapy.

## Competing interests

The authors declare that they have no competing interests.

## Authors’ contributions

CSD, LVD and MF conceived of the study and drafted the manuscript. BP carried out patients’ monitoring and helped to draft the manuscript. IE, AK and EW performed the experiments and summarized primary data. All authors read and approved the final manuscript.

## Supplementary Material

Additional file 1: Table S1 Demographic characteristics of patients undergoing radiation therapy. **Table S2.** Demographic characteristics of patients undergoing radiation therapy (Summary). **Figure S1. **Correlational analysis of mean γ-H2AX and 53BP1 foci counts from 500 nuclei per sample. Non-irradiated and irradiated with 0.5 and 2 Gy lymphocytes were fixed 30 min or 24 post-IR. The expression of both proteins was analyzed simultaneously at each time and IR points for n=30 blood samples (BC39-BC69). **Figure S2.** Correlation between the γ-H2AX/53BP1 foci expression and tumor staging. Peripheral lymphocytes were prepared from the blood samples derived from breast cancer patients. Foci counting for γ-H2AX and 53BP1 foci were performed in non-irradiated and irradiated with 0.5 Gy samples 30 min post-IR derived from 57 and 39 patients, respectively. Click here for file
