# Supplementary material for: Radiosensitivity in breast cancer assessed by the histone γ-H2AX and 53BP1 foci
Source: Radiat Oncol. 2013 Apr 24;8:98. doi: 10.1186/1748-717X-8-98 (PMC3653697; doi:10.1186/1748-717X-8-98)
Supplement: Additional file 1: Table S1 — Demographic characteristics of patients undergoing radiation therapy. Table S2. Demographic characteristics of patients undergoing radiation therapy (Summary). Figure S1. Correlational analysis of mean γ-H2AX and 53BP1 foci counts from 500 nuclei per sample. Non-irradiated and irradiated with 0.5 and 2 Gy lymphocytes were fixed 30 min or 24 post-IR. The expression of both proteins was analyzed simultaneously at each time and IR points for n=30 blood samples (BC39-BC69). Figure S2. Correlation between the γ-H2AX/53BP1 foci expression and tumor staging. Peripheral lymphocytes were prepared from the blood samples derived from breast cancer patients. Foci counting for γ-H2AX and 53BP1 foci were performed in non-irradiated and irradiated with 0.5 Gy samples 30 min post-IR derived from 57 and 39 patients, respectively. [file 1748-717X-8-98-S1.doc]

**Table S1**

Demographic characteristics of patients undergoing radiation therapy

| *Subject* | *Age (years)* | *Sex* | *Clinical status with respect to cancer, skin reaction to RT* | *Tumor  stage* | *Alcohol  consumption* | *Tobacco  consumption* |
| --- | --- | --- | --- | --- | --- | --- |
|  |  |  |  |  |  |  |
|  |  |  |  |  |  |  |

**Breast cancer patients**

| BC001 | 59 | F | **BC, grade 3** | 2 | moderate | never |
| --- | --- | --- | --- | --- | --- | --- |
| BC002 | 63 | F | BC, grade 1 | 1 | moderate | never |
| BC003 | 45 | F | BC, grade 0 | DCIS | never | never |
| BC004 | 73 | F | **BC, grade 3** | 1 | never | never |
| BC005 | 57 | F | BC, grade 1 | 1 | never | never |
| BC006 | 50 | F | BC, grade 1 | 1 | not specified | not specified |
| BC007 | 63 | F | BC, grade 2 | 1 | moderate | never |
| BC008 | 70 | F | BC, grade 2 | 1 | never | never |
| BC012 | 61 | F | **BC, grade 3** | 2 | never | never |
| BC013 | 55 | F | **BC, grade 3** | 1 | never | ex-smoker |
| BC014 | 54 | F | BC, grade 2 | 1 | not specified | never |
| BC020 | 52 | F | BC, grade 1 | 1 | moderate | never |
| BC023 | 51 | F | BC, grade 1 | 2 | never | never |
| BC025 | 41 | F | BC, grade 0 | 2 | not specified | ever |
| BC026 | 63 | F | BC, grade 1 | 1 | moderate | never |
| BC027 | 71 | F | BC, grade 1 | 1 | ever | never |
| BC028 | 42 | F | BC, grade 0 | 1 | never | never |
| BC029 | 74 | F | BC, grade 1 | 2 | moderate | never |
| BC030 | 46 | F | BC, grade 2 | 1 | moderate | ex-smoker |
| BC031 | 56 | F | BC, grade 2 | 1 | moderate | never |
| BC032 | 44 | F | BC, grade 2 | 2 | never | never |
| BC033 | 41 | F | **BC, grade 3** | 1 | never | ever |
| BC034 | 38 | F | BC, grade 2 | 1 | moderate | never |
| BC035 | 77 | F | BC, grade 2 | 2 | never | never |
| BC036 | 70 | F | BC, grade 1 | 1 | never | never |
| BC037 | 64 | F | BC, grade 0 | 1 | moderate | never |
| BC038 | 73 | F | BC, grade 1 | 2 | not specified | not specified |
| BC039 | 55 | F | BC, grade 1 | 1 | moderate | never |
| BC040 | 77 | F | BC, grade 2 | 1 | never | never |
| BC041 | 54 | F | BC, grade 1 | 2 | moderate | never |
| BC042 | 49 | F | BC, grade 1 | 1 | never | never |
| BC043 | 62 | F | BC, grade 2 | 2 | never | never |
| BC044 | 59 | F | BC, grade 1 | 1 | moderate | never |
| BC045 | 51 | F | BC, grade 1 | DCIS | moderate | ever |
| BC046 | 62 | F | BC, grade 1 | 1 | moderate | never |
| BC047 | 30 | F | BC, grade 2 | 1 | not specified | never |
| BC048 | 71 | F | BC, grade 2 | 1 | never | never |
| BC049 | 46 | F | BC, grade 2 | 1 | never | never |
| BC050 | 48 | F | **BC, grade 3** | 1 | never | ever |
| BC051 | 57 | F | BC, grade 1 | 1 | not specified | not specified |
| BC052 | 62 | F | BC, grade 2 | 1 | moderate | never |
| BC053 | 68 | F | BC, grade 2 | 2 | moderate | never |
| BC054 | 37 | F | BC, grade 1 | 2 | never | never |
| BC055 | 67 | F | BC, grade 1 | 1 | moderate | never |
| BC057 | 55 | F | BC, grade 1 | 1 | moderate | never |
| BC058 | 62 | F | BC, grade 1 | 1 | moderate | not specified |
| BC059 | 55 | F | BC, grade 1 | 2 | moderate | ever |
| BC060 | 65 | F | BC, grade 2 | 1 | never | never |
| BC061 | 54 | F | BC, grade 2 | 3 | never | never |
| BC062 | 53 | F | BC, grade 2 | 4 | moderate | never |
| BC063 | 69 | F | BC, grade 1 | 1 | never | never |
| BC064 | 81 | F | BC, grade 1 | 1 | never | never |
| BC065 | 38 | F | BC, grade 2 | 2 | never | never |
| BC066 | 77 | F | BC, grade 1 | 1 | never | never |
| BC067 | 48 | F | BC, grade 1 | 2 | moderate | never |
| BC068 | 49 | F | BC, grade 2 | 1 | moderate | never |
| BC069 | 51 | F | BC, grade 1 | DCIS | never | never |
| Mean | 57 |  |  |  |  |  |
| ± SD | 12 |  |  |  |  |  |

**Table S2**

Demographic characteristics of patients undergoing radiation therapy (Summary)

*Patient clinical details Value*

No. of patients 57

Mean age 57 (range 38-57)

No. of males 0

No. of females 57

Smoking 7/57

Alcohol consumption 27/57

Tumor stage
DCIS 3
T1 37
T2 15
T3 1
T4 1

RTOG skin reaction**^#^** grade
0 4
1 27
2 20
3 6
4 0

**^#^**Early skin reaction was controlled at the end of RT according RTOG (Radiation Therapy Oncology Group) score (Cox et al., 1995). RTOG grade: 1 - follicular, faint or dull erythema, dry desquamation; 2 - tender or bright erythema, moderate edema; 3 - confluent, moist desquamation, pitting edema; 4 - ulceration, haemorrhage, necrosis.

|  |
| --- |
| **Fig. S1**. Correlational analysis of mean γ-H2AX and 53BP1 foci counts from 500 nuclei per sample. Non-irradiated and irradiated with 0.5 and 2 Gy lymphocytes were fixed 30 min or 24 post-IR. The expression of both proteins was analyzed simultaneously at each time and IR points for n=30 blood samples (BC39-BC69). |

|  |
| --- |
| **Fig. S2.** Correlation between the γ-H2AX/53BP1 foci expression and tumor staging. Peripheral lymphocytes were prepared from the blood samples derived from breast cancer patients. Foci counting for γ-H2AX and 53BP1 foci were performed in non-irradiated and irradiated with 0.5 Gy samples 30 min post-IR derived from 57 and 39 patients, respectively. |
